# Expitope 2.0: a tool to assess immunotherapeutic antigens for their potential cross-reactivity against naturally expressed proteins in human tissues

**DOI:** 10.1186/s12885-017-3854-8

**Published:** 2017-12-28

**Authors:** Victor Jaravine, Anja Mösch, Silke Raffegerst, Dolores J. Schendel, Dmitrij Frishman

**Affiliations:** 1 Department of Bioinformatics, Wissenschaftszentrum Weihenstephan, Technische Universität München, Freising, 85354 Germany; 2 Medigene Immunotherapies GmbH, a subsidiary of Medigene AG, Planegg, 82152 Germany; 3 St Petersburg State Polytechnical University, St Petersburg, 195251 Russia

**Keywords:** Cancer, Immunotherapy, Tumor immunology, Cross-reactivity, T cell epitope, Immunoinformatics, Tumor antigen expression

## Abstract

**Background:**

Adoptive immunotherapy offers great potential for treating many types of cancer but its clinical application is hampered by cross-reactive T cell responses in healthy human tissues, representing serious safety risks for patients. We previously developed a computational tool called Expitope for assessing cross-reactivity (CR) of antigens based on tissue-specific gene expression. However, transcript abundance only indirectly indicates protein expression. The recent availability of proteome-wide human protein abundance information now facilitates a more direct approach for CR prediction. Here we present a new version 2.0 of Expitope, which computes all naturally possible epitopes of a peptide sequence and the corresponding CR indices using both protein and transcript abundance levels weighted by a proposed hierarchy of importance of various human tissues.

**Results:**

We tested the tool in two case studies: The first study quantitatively assessed the potential CR of the epitopes used for cancer immunotherapy. The second study evaluated HLA-A*02:01-restricted epitopes obtained from the Immune Epitope Database for different disease groups and demonstrated for the first time that there is a high variation in the background CR depending on the disease state of the host: compared to a healthy individual the CR index is on average two-fold higher for the autoimmune state, and five-fold higher for the cancer state.

**Conclusions:**

The ability to predict potential side effects in normal tissues helps in the development and selection of safer antigens, enabling more successful immunotherapy of cancer and other diseases.

**Electronic supplementary material:**

The online version of this article (doi:10.1186/s12885-017-3854-8) contains supplementary material, which is available to authorized users.

## Background

The principles of how the immune system can optimally control infections and early stages of cancer underpin the development of immunotherapies. Among these approaches, adoptive transfer of antigen-specific T cells is emerging as a particularly attractive form of immunotherapy to treat patients with more advanced stages of cancer and unresolved infectious diseases. This approach utilizes transfer of tailored antigen-specific immune T cells and provides the possibility of clinically efficient treatment of infectious diseases and human malignancies [1].

One major stumbling block precluding wider application of adoptive immunotherapy is the occurrence of adverse effects of off-target cross-reactivity (CR), which may result in significant, even lethal, toxicity. The cause of toxicity is a hyper-activated T cell response with reactivity directed against normal tissue [2]. Immune CR arises when T cells recognizing a selected target epitope are transferred back to the patient and exhibit recognition of self-epitopes in non-cancerous tissues. On the molecular level this effect is usually the consequence of a high degree of sequence similarity between the target and the self-epitopes, resulting in the binding of a stable self-peptide-MHC complex to the T cell receptor (TCR) and, consequently, cross-activation of unwanted autoimmune T cell responses [3]. Depending on the sequence similarity there can be on-target/off-tumor or off-target recognition. The former is directed against the identical epitope that is also present in a non-cancerous tissue, while the latter is directed against a similar epitope also present in a healthy tissue. The ability to predict the scope and extent of on- and off-target effects can help in selection of safer antigens, and consequently enable more successful immunotherapy treatment [4].

A computational strategy for the prediction of potential peptide-HLA cancer targets and evaluation of the likelihood of off-target toxicity for the targets was developed by Dhanik et al. [5]. The strategy utilizes a sequence-based algorithm similar to the one used in our previous studies [6] and in our current work, but it is not available as a web-service.

We have developed the Expitope server as a tool to assess epitope expression in various tissues (freely accessible at http://webclu.bio.wzw.tum.de/expitope2). Expitope incorporates the most recent genome-wide information, including protein sequences and protein abundance data across various tissues and cell lines. It enables researchers to screen their epitopes in silico for potential CR in human tissues, before moving their therapeutic candidates into clinical trials.

## Approach

CR to an immunotherapeutic epitope may arise if a protein normally expressed in healthy cells is cleaved by one of the proteasomes to produce a peptide with an amino acid sequence that is similar to the given epitope. Another prerequisite for CR is the presentation of the natural epitope by major histocompatibility complex class I molecules (MHC-I) in various tissues. We model this process by the method described by Keşmir et al. [7]. To quantitatively assess the natural occurrence of epitopes, we use experimental data on gene expression and abundance of proteins in which the epitopes are present. The methods are described in detail in our previous publication [8] on the iCrossR tool, which has been merged into the current version 2.0 of Expitope. The iCrossR project’s aim was to perform a quantitative characterization study of all MHC-I epitopes listed in the cancer immunotherapy database. A new feature of Expitope 2.0 is the calculation of the tissue-weighted cross-reactivity (CR) indices. Below we test the approach and provide information on the new data sources and a new tissue-weighted CR-index formula.

## Material and Implementation

### Gene and protein expression data

The previous version 1.0 of Expitope [6] assessed the expression of human antigens based on one combined gene expression database [9] and the Illumina Body Map database [10]. Interestingly, HLA-typing of samples from the Illumina Body Map and Wang et al. [9] showed that the tissues used for expression analysis are most likely derived from the same individual except for seven brain samples [11]. In order to avoid data redundancy with the new Illumina Body Map database, we now only use the brain expression data from Wang et al. [9]. The new version 2.0 of Expitope incorporates three gene expression and four protein abundance datasets (Table 1). It should be noted that in contrast to the PaxDB and Human Proteome Map datasets, which contain ppm values, the Human Protein Atlas data has been generated by immunohistochemistry, which makes the accuracy of the data dependent on the specificity of the antibodies used. The values range from 0 to 3, indicating no detectable expression (0) up to high expression (3).

**Table 1:** Sources of gene expression and protein abundance data

Data source	ID	Name	Number of tissues	Type	References
PaxDB	Pax4	PaxDB v4.0	22	Protein abundance	[24]
Expression Atlas	E-Prot-3	Human Protein Atlas	44	Protein abundance	[25, 26]
Expression Atlas	E-Prot-1	Human Proteome Map	23	Protein abundance	[25, 27]
Expression Atlas	E-Mtab-513	Illumina Body Map	16	Gene expression	[10, 25]
Expression Atlas	E-Mtab-5214	GTEx	53	Gene expression	[25, 28]
Wang et al. 2008	Wang	Wang 2008	7	Gene expression	[9]
Expression Atlas	E-Mtab-3358	FANTOM5 RIKEN	56	Gene expression	[25, 29]

### IEDB datasets

We selected four groups of peptides (Table 2) from the Immune Epitope Database (IEDB) [12], containing a total of 1720 epitopes of 7-25 amino acids in length (Additional file 1: Table S1, Additional file 2: Table S2, Additional file 3: Table S3, Additional file 4: Table S4). The selection for all groups was restricted to the following tags: ’human HLA-A*02:01’, ’Linear Epitopes’, ’Positive Assays only’, ’T cells Assays’, ’MHC ligand Assays’, ’No B-cell assays’, ’Host: Homo Sapiens (Human)’, from which the selection was further restricted for each of the four groups using the tag corresponding to a disease state of the host (column 3 of Table 2).

**Table 2:** Four epitope groups from the IEDB database

Group	ID in IEDB	Disease state of host	Number of entries	Peptide length range (average)
1	DOID:0050117	Infectious diseases	588	8-20 (9)
2	DTREE_00000014	Healthy (no disease)	461	8-25 (10)
3	DOID:417	Autoimmune diseases	155	8-21 (10)
4	DOID:162	Cancer	516	7-25 (11)

### Identification of natural epitopes

Amino acid sequences of epitopes were matched against the RefSeq database [13] of all naturally occurring human protein sequences, including annotated isoforms, downloaded from the National Center for Biotechnology Information (NCBI). The matching procedure yields a list of protein segments, which we call “natural epitopes” (NEs). Potential immunogenicity of each NE was calculated using the formula developed by Keşmir et al. [7], which combines the predicted scores for proteasomal cleavage, TAP affinity and MHC-binding predictions. The quantitative score *Q* of epitope presentation on MHC-I is defined as:

1 $$ Q = P_{CL}/\left(A_{TAP}*A_{MHC}\right)  $$

where *P*_*CL*_ is the proteasomal cleavage probability, while *A*_*TAP*_ and *A*_*MCH*_ are the IC_50_-affinities to the transporter molecule associated with antigen processing (TAP) and to the MHC complex, respectively. Lower values for *A*_*TAP*_ and *A*_*MHC*_ correspond to higher predicted affinities, as IC_50_-affinity is defined as a dose of peptide that displaces 50% of a competitive ligand.

### Calculation of the tissue weighted CR-index

In this version, we modified the CR-index calculation formula [8] to include tissue weighting, reflecting the perceived importance of different tissue types in the human body. For each database, the tissue profile *S(t)* for a given epitope was calculated as follows:

2 $$ S(t) = \sum_{k=0}^{K} \left\{ v(k) \cdot \log_{10} \left[ \sum_{i=1}^{M(k)} a(i,t) \right] \right\}  $$

where *k* is the allowed number of mismatches and *K* is the maximal *k*; *t* is the tissue index in a given database of *T* tissues; *i* is the running index in the list of matching NEs for each *k*, and *M(k)* is the size of the list; *v(k)* is the normalized mismatch weight, and *a(i,t)* is the protein or transcript abundance in the tissue *t* corresponding to the *i*-th NE. The sum over *i* includes only the unique NEs that have the scores *Q(i)* (Equation 1) above a chosen threshold. The normalized mismatch weight is calculated as *v(k) = (1/P(k))/* $\sum $_k_(1/P(k)), where *P(k)* is the probability of finding a random peptide of length *l* with *k* mismatches in our protein sequence database of the total length of *N*=6.5e7 amino acids, *P(k) = 1-(1-0.05* ^*l-k*^ *)* ^*N-l+1*^. For example, for a peptide of length 9, the mismatch weights are: *v*(*k*=0,1,2,3) = 0.95, 0.0475, 0.0023, 0.0002.

The weighted CR-index is defined as a tissue-weighted average of the tissue profiles *S(t)*:

3 $$ I_{CR} = \frac{1}{\sum_{t}^{T} w(t)} \sum_{t}^{T} w(t) S(t)   $$

where *w(t)* represents the weight assigned to the tissue type *t* (Table 3). The *I*_*CR*_ index error is obtained as one standard deviation from the mean upon bootstrapping, which involves repeating index calculation 10 times using 90% of randomly subsampled data. The weight values range between 0 and 1, with the weight of 1 corresponding to the most vital organs and systems according to the Sequential Organ Failure Assessment (SOFA) score used to evaluate the condition of patients in Intensive Care Units (ICUs) [14]. The second highest weight of 0.8 is assigned to tissues that belong to vital organs where a failure does not immediately threaten a patient’s life. A weight of 0.5 is assigned to tissues where CR is not necessarily life threatening, but can nevertheless cause severe complications. The second lowest weight of 0.3 refers to tissues and organs that can be surgically removed without major complications. Finally, the weight of 0 was assigned to irrelevant tissues such as testis, where expression of an antigen does not cause an immune response, as well as to the tissues that are only present during pregnancy and other samples that do not correspond to healthy human tissue, e.g. cancer cell lines.

**Table 3:** Weight values and categorization of tissue types

*Consequence*	*Damage immediately life*	*Damage life threatening*	*Damage not immediately*
	*threatening*		*life threatening*
*Weight*	1	0.8	0.5
*Tissues*	Lung/Respiratory system	Digestive system	Urinary bladder
	Brain/Nervous system	(except appendix)	Various glands
	Blood/Immune system	Soft tissue	Prostate
	Heart		Skin
	Kidney		Eye ^a^
	Liver		
*Consequence*	*Damage not life*	*Tissue not affected*	
	*threatening*		
*Weight*	0.3	0	
*Tissues*	Reproductive organs	Cancer cell lines	
	Mammary tissue	Testis	
	Tonsils	Fetal tissue	
	Appendix		
	Gall bladder		
	Spleen		

^a^The weight for eye tissue is set to 0.5, as T cells are able to infiltrate it [30]

Consequently, large *I*_*CR*_ values may indicate potentially life-threatening CR of the epitope. The higher the number of hits to different NEs that are close in sequence to a therapy peptide, and their total abundance/expression levels in the tissues with high weights, the higher is the probability of CR. Higher thresholds for *Q* correspond to choosing a higher probability of the selected natural epitope to be immunogenic, while the parameter *K* controls the sequence similarity: exact match (*K*=0) for prediction of on-target/off-tumor recognition, and *K* > 0 for off-target recognition. The values of these parameters can be set by Expitope users. In this work, we chose *K*=1, i.e. up to one mismatch in amino-acid sequence, and two thresholds for *Q*: 0.02 corresponding to top 10% immunogenic NEs found for all epitopes in this study, and 1e-4 corresponding to top 50% of the NEs, i.e. top-scored for proteasomal cleavage, TAP transport and MHC-I binding. However, calculation of the indices with the numbers of mismatches *K*=[0,3] and the combined scores *Q*=0.02, 1e-4, 1e-5 gave very similar results (Additional file 5: Tables S5-S7; Figure S2).

While a high *I*_*CR*_ means that severe complications are expected for a target epitope, its low value hints towards minor or non-life-threatening side effects. An index greater than zero always means that there is some expression present that should be investigated in detail. The index is only an estimate, which does not take into account many patient-specific factors, and therefore should not be used as the sole measure for making decisions. As the tissue classification is not exhaustive and not all organs are completely represented by the tissue types of which they consist, a high expression value in a low rated tissue could correspond to a tissue type not covered, but also present in other more vital organs. Nonetheless, the weighted index offers a short summary of the rather extensive result tables that are produced by Expitope 2.0, and contain individual expression values for each tissue and all NEs. Therefore, the weighted index allows for quick rejection of target epitopes that are likely to cause severe side effects caused by CR.

The *I*_*CR*_ indices were calculated with the default parameters (except *Q* and *K*) for each peptide and each database using Eq. 3, and were averaged over the seven databases to give the average *I*_*CR*_ indices for each peptide. For the plots the *I*_*CR*_ indices are averaged for all peptides in each group.

### Web server

Expitope 2.0 is a web application that can be easily used by the researchers inexperienced in bioinformatics, especially from the immunotherapy domain. There is no login requirement to the website and user IP addresses are not stored. Multiple clients can connect to the server, and concurrent clients are served one query at a time. The jobs are submitted to high-performance computational infrastructure. The results are displayed once they are ready; alternatively the user can return to the results later, using the session URL. It is also possible to download the results as a spreadsheet to be used with Microsoft Excel or similar software. This allows to sort and filter the results according to individual criteria, e.g. for sorting epitopes by binding affinity predicted by netMHC.

**Fig. 1:**
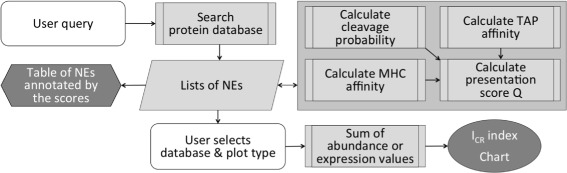
Workflow of the Expitope 2.0 web server

The workflow of Expitope is shown in Fig. 1. The user inputs a peptide sequence and specifies parameters for sequence matching and for the computation of MHC class I binding affinity via the html forms displayed in a web-browser (white). The server performs the search for natural epitopes (NEs) and calculates their *Q* scores. Computations are performed by the client process at the backend of the server (large gray rectangle). Results are returned to the user in the form of text files and graphical visualizations (dark gray). The user selects a particular database and a plot type for visualization (white). The parameters that can be changed by users in the forms have the following default values: the TAP weight is 0.2, the cleavage threshold is 0.7, the *Q* score threshold is 1e-4 and the number of mismatches is 2.

## Results and discussion

### Known cross-reactive epitopes

For the first version of the Expitope web server, the MAGEA3 epitope EVDPIGHLY was tested that had been associated with cross-reactivity caused by the TCR recognizing an epitope with four mismatches derived from titin, which is expressed in heart muscle tissue [6, 15]. We were able to reproduce these findings by using Expitope 2.0 with default the parameters except for allowing up to four mismatches and additionally, the newly added protein databases showed an even clearer result with values of 2.98e+03 ppm (PaxDB) and 2.86e+03 ppm (Human Proteome Map) and the maximum value of 3 for the Human Protein Atlas. Another case of observed cross-reactivity has been a TCR recognizing the MAGEA3/MAGEA9 epitope KVAELVHFL [16]. Expitope 2.0 with the default parameters finds this and all other epitopes from various members of the MAGE family the TCR was able to detect. This includes one epitope of MAGEA12, which was found to be expressed in brain where it led to cross-reactivity. We found expression values of 0.2 FPKM and less but no protein expression for MAGEA12, which is also not contained in the Human Protein Atlas and Human Proteome Map. This demonstrates the importance of taking even small amounts of expression into account when assessing potential cross-reactivity and also comparing the results obtained from all databases, especially for crucial tissues like heart, brain and lung.

### Case studies

#### Cancer immunity peptides

Here we provide an overview of our previous study [8], where we analyzed short (8-15 amino acids) peptide sequences from the Cancer Immunity Peptide Database [17] as well as peptides of viral origin. The CR-index calculation was based only on the PaxDB protein abundance database and without tissue weighting.

The peptide dataset consisted of four groups of currently known human MHC class I epitopes including: mutation antigens displayed by tumor cells (40 peptides, group A), cancer-testis (CT) antigens (67 peptides, group B), differentiation antigens (57 peptides, group C), and overexpressed proteins (94 peptides, group D). In addition, 89 epitopes originating from viral sources (group E) were investigated. When matched exactly, the group of “mutation” antigens produced no hits to the proteins normally expressed in human tissues, since the epitopes of the group have sequences that originated from mutations of normal human protein sequences. The second validation is from the CT antigens, which at small numbers of mismatches (0-1), showed few matches to proteins expressed in the majority of human tissues, with the expected exception of ovary/testis, where multiple hits were found. The hit patterns were very similar for all epitopes of this group. This is exactly as expected, since CT antigens are expressed mostly in these two tissues. In contrast to the results for groups A and B, the antigens of the groups C and D showed more hits, both for exact matches and for high numbers of mismatches. This is also as expected as the proteins containing the epitopes are expressed in a wide variety of normal tissues. Finally, the epitopes originating from the viral sources showed noticeably fewer matches to the human proteins compared to the cancer peptides.

#### IEDB epitopes

We sought to assess quantitatively the extent of potential “background” CR of the epitopes derived from the host individuals having different disease states - ranging from healthy to cancer. Such background CR is not caused by one single therapy but accumulates due to many factors, including an unknown history of diseases.

The *I*_*CR*_ indices of individual epitopes calculated across the seven databases used in this study are highly correlated, since for each database they are obtained by summation of the abundance (or expression) values for the same proteins. There are high correlations between the *I*_*CR*_ values computed for the peptides using the three abundance databases as well as between the *I*_*CR*_ values derived from the four expression databases (data not shown). Similarly, the correlations between the abundance and expression indices are high (Additional file 5: Figure S1), with the Pearson’s coefficients in the range 0.94-0.96. Averaging of the indices allows one to obtain a more accurate prediction of CR due to increased signal-to-noise ratio, as the databases are derived from different data sources.

**Fig. 2:**
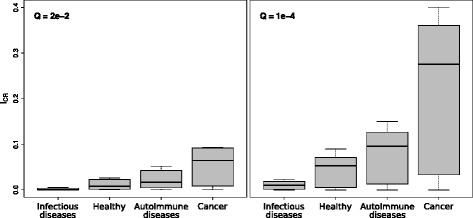
The *I*_*CR*_ indices for the four IEDB peptide groups (Table 2), obtained by averaging over the seven databases listed in Table 1. *Q*=2e-2 (left), *Q*=1e-4 (right), with up to one mismatch (*K*=1). Thick black line: median; gray: the lower and the upper quartiles (25th and 75th percentiles); upper and lower whiskers: highest and lowest values

Figure 2 shows the *I*_*CR*_ indices for the four epitope groups described in Table 2 (group *I*_*CR*_ indices before averaging by databases can be found in Additional file 5: Tables S5-S7). The indices for the epitopes computed from 10% top-scoring NEs (*Q*=0.02, Fig. 2 left) are on average 3-times lower, compared to those from 50% top-scoring NEs (*Q*=1e-4, Fig. 2 right), corresponding to lower numbers of matching NEs. Higher thresholds for *Q* correspond to a higher probability of the selected NEs to be immunogenic. It has been reported that the top-scoring 7-10% epitopes identified by the immunogenicity prediction methods have 85% probability of being immunogenic [18]. In this work we have chosen two thresholds of 10% and 50% of sequence matches. The rationale for this choice was to ensure a low amount of false positives in the immunogenicity prediction for the 10% *I*_*CR*_ index, and to compare it with the 50% value containing medium to high immunogenic peptides. Two groups - ‘Infectious diseases’ and ‘Healthy’ - have average indices close to zero on both plots, indicating low amounts of cross-reactive epitopes in the critical tissues. The groups ‘Autoimmune diseases’ and ‘Cancer’ exhibit approximately 2- to 5-fold higher average index values compared to the ‘Healthy’ group, in each plot respectively, corresponding to considerably higher presentation level of the cross-reactive peptides in these states.

The interpretation of these results is as follows. The epitopes in the ‘Infectious diseases’ group are derived from non-human organisms rather than from human hosts. Thus, compared to the epitopes from the other three groups, which are of human origin, a lower *I*_*CR*_ index is expected, implying low sequence identity to the host and thus a low probability of CR. The slightly elevated index for the ‘Healthy’ group is most likely due to the presence of common pathogens (such as Herpes simplex virus or Epstein-Barr virus) mimicking human sequences, an immune escape strategy known as immune camouflage [19]. A higher *I*_*CR*_ for the ‘Autoimmune’ group compared to the ‘Healthy’ group is not surprising, as autoimmunity is a response of the human body’s immune system directed against human proteins overexpressed or aberrantly presented in healthy tissues. For example, multiple sclerosis, the most frequently occurring disease in this group, is due to autoimmunity to the myelin basic protein (MBP), expressed in the tissues of the central nervous system [19]. Other epitopes in this group with very high index values are derived, e.g. from the proteins actin, myosin-9, septin-2 and vimentin, which are normally expressed in various tissues. Normally, peripheral T cells are trained to recognize pathogen-derived epitopes and ignore self-antigens, however some T cells escape this selection and are able to recognize self-antigens, thus initiating an autoimmune response and becoming self-reactive. Consequently with respect to autoimmunity, the term CR is defined as the recognition by T cell TCRs of many different peptide antigens, presented by the HLA of an individual [20], which can also be referred to as cross-recognition.

The significantly higher CR index for the cancer group compared to the other three groups indicates a presence of a high background level of CR when targeting cancers. Cancer epitopes originate either from wild-type proteins overexpressed in tumors, or as a result of cancer-specific mutations in the genes, named neoepitopes. On average, neoepitopes have lower similarity to self-antigens compared to the wild-type cancer epitopes, thus potentially are less cross-reactive. Since T cells with TCRs binding to self-antigens are negatively selected in the thymus, there will generally be a lack of the T cells that can fight tumors, producing overexpressed wild-type proteins. In contrast, the cancers producing neoepitopes can be effectively controlled by the immune system provided that suitable T cells are available. Thus, different types of cancer produce epitopes of varying cross-reactivity, which explains the larger variance seen in Fig. 2 for the cancer group compared to the other groups.

High *I*_*CR*_ for the ‘Autoimmune disease’ and ‘Cancer’ groups may also be due to an activated state of the immune system, when immunoproteasomes create larger amounts of immunogenic (in comparison to standard proteasomes) epitopes, including those from the residuals of normal cells killed by the immune system [21]. In addition, disruption of the normal functioning of the ubiquitin proteasome system may result in creation of abnormally presented immunogenic epitopes, leading to many types of disorders, including malignancies, neurodegenerative diseases and systemic autoimmunity [22, 23].

Thus, multiple reasons for a high variability in presented CR epitopes appear to exist depending on the host disease state. This CR, which we tentatively call “background” CR, is independent of any immune therapy. Clearly, a collection of epitopes present in a particular individual is different from our datasets obtained from the IEDB database. Likely, it will include only a subset of the peptides, but a statistical distribution in many patients may exhibit a pattern similar to the one reported in this work. Eventually, it remains to be seen if there can be any interference between the background CR and the CR invoked by a therapy, but both types are important to assess the safety of the therapy.

## Conclusion

It is a long-standing dream of many medical practitioners to use the immune system for effective treatment and permanent cure of human disease conditions. With the number of tested and approved immunotherapies growing, evidence of the side effects associated with the current therapies also increased. Consequently, therapy developers require reliable tools for predicting unwanted cross-reactions.

The Expitope web tool for predicting CR of T cell epitopes is based on experimental protein abundance and expression data obtained from a growing number of publicly available databases. We demonstrate its performance for a large number of epitopes detected in the human organism for various cancer types and at various diseases states, ranging from healthy to cancer. The results of our study of Cancer Immunity Peptides [8] showed that the currently known cancer epitopes display a very large CR variability across a range of tissues. Our predictions are in close agreement with the results of several clinical studies, with the CR indices being high in the tissues where actual side effects have been reported, and close to zero for no side-effects. Thus, Expitope enables researchers to assess potential side effects of their selected antigens for therapy and to identify specific human tissues where such side effects could be expected. Since any immunotherapy can cause side effects, we suggest using this tool at both early and late stages of a therapy development process. CR index values calculated by Expitope can serve as an estimate of the amount of potential CR for *in silico* assessment of immunotherapeutic strategies.

For the first time we demonstrate that there is a high variation in the CR of peptides presented at different disease states of the host: it is on average 2-fold higher for individuals with an autoimmune state and 5-fold higher for individuals with cancer in comparison to individuals in an apparent healthy state. Presumably, a similar background CR may exist prior to an immune therapy, which may differ by the host disease state. Since the human organism negatively pre-selects T cells binding to self-antigens, there will be a small number or no T cells fighting disease tissue cells marked by highly cross-reactive epitopes. Consequently, the similarity of presented epitopes to self-antigens is an obstacle for disease elimination both for the organism itself and for immunotherapy. Thus, therapy developers should consider the possibility of background CR interfering with a therapy.

## Availability and requirements

**Project name:** Expitope 2.0

**Project home page:** http://webclu.bio.wzw.tum.de/expitope2

**Operating system(s):** Platform independent

**Programming language:** Java, JavaScript

**Other requirements:** Web browser

**License:** None (free to use for academic purposes)

**Any restrictions to use by non-academics:** None

## Additional files

Additional file 1 TableS1_InfectiousDisease. Comma-separated table containing Table S1. (CSV 80 kb)

Additional file 2 TableS2_Healthy. Comma-separated table containing Table S2. (CSV 67 kb)

Additional file 3 TableS3_AutoimmuneDisease. Comma-separated table containing Table S3. (CSV 20 kb)

Additional file 4 TableS4_Cancer. Comma-separated table containing Table S4. (CSV 67 kb)

Additional file 5 Suppl-Material. Microsoft Word file containing Figures S1 and S2 and Tables S5-S7. (DOCX 191 kb)

## Abbreviations

CR: Cross-reactivity
CT: Cancer-testis
HLA: Human leucocyte antigen
IEDB: Immune epitope database
MHC: Major histocompatibility complex
NE: Natural epitope
TAP: Transporter associated with antigen processing
TCR: T cell receptor
