# Supplementary material for: Expitope 2.0: a tool to assess immunotherapeutic antigens for their potential cross-reactivity against naturally expressed proteins in human tissues
Source: BMC Cancer. 2017 Dec 28;17:892. doi: 10.1186/s12885-017-3854-8 (PMC5745885; doi:10.1186/s12885-017-3854-8)
Supplement: Supplementary file 5 — Suppl-Material. Microsoft Word file containing Figures S1 and S2 and Tables S5-S7. (DOCX 191 kb) [file 12885_2017_3854_MOESM5_ESM.docx]

## SUPPORTING DATA

Supporting data files contain the following information: the lists of the epitopes obtained from IEDB (in csv format) for the four groups (Tables S1-S4: “Infections”, “Healthy”, “Autoimmune”, “Cancer”); the I_CR_ indices computed for each epitope and seven databases for three combined score values (Q=2e-2, 1e-4, 1e-5) and for four mismatch numbers (k=0,1,2,3) (text files), as well as given below Tables S5-S7 as text files. The files can be downloaded from <http://webclu.bio.wzw.tum.de/expitope2/SupplMaterialData.tgz>


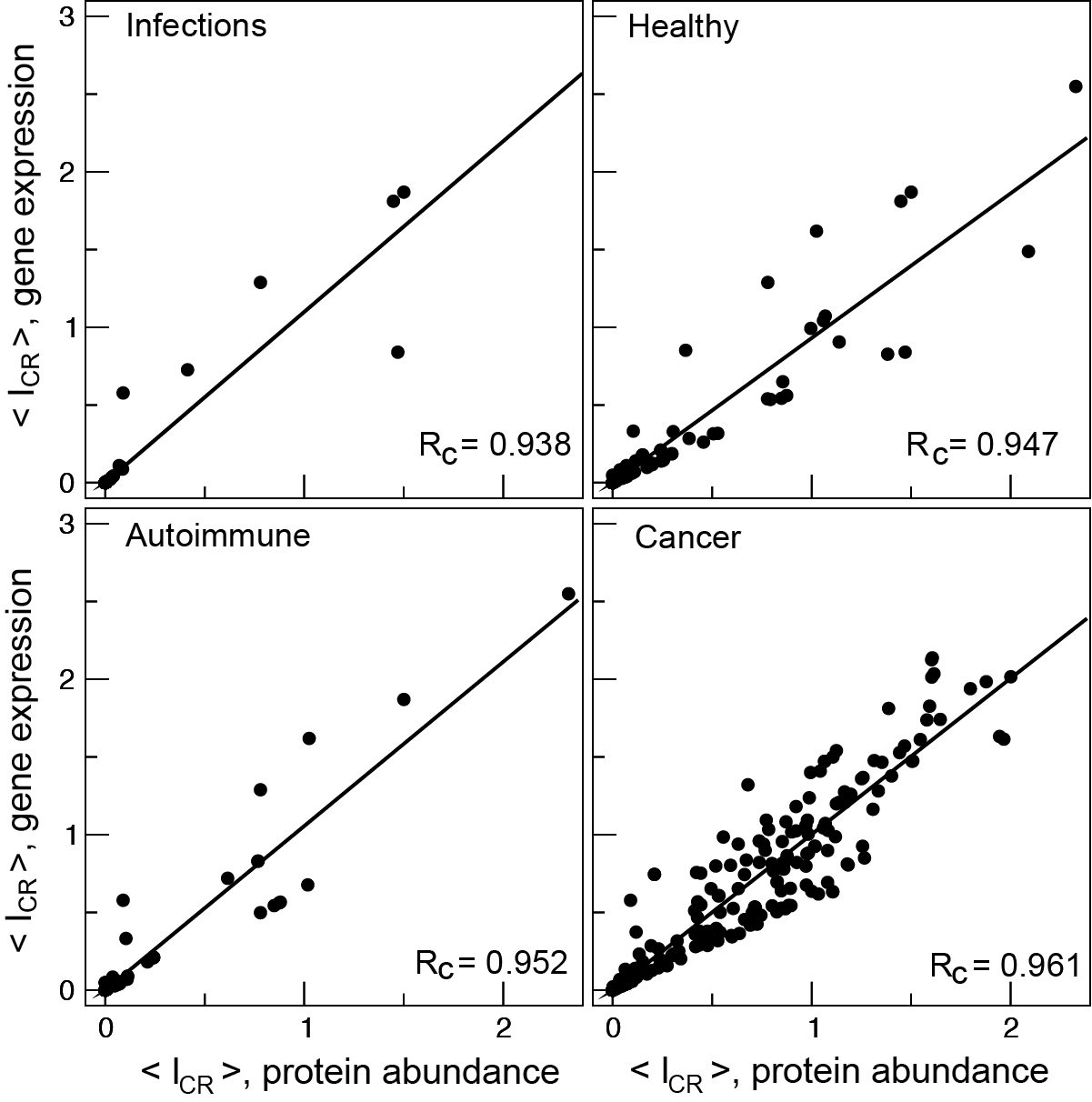


Fig. S1. Comparison of the <I_CR_> indices calculated based on protein abundance and gene expression data for the four groups of epitopes from the IEDB database: Infections, Healthy, Autoimmune, Cancer. Pearson’s correlation coefficients R_c_ are shown. <I_CR_> values based on protein abundance and gene expression are calculated by simple averaging of the I_CR_ indices over the abundance (#1-3, Table 1) and expression (#4-7, Table 1) databases. Q=1e-4, k=3.

**Tables S5-S7.** The <I_CR_> values for the four groups of epitopes from the IEDB and the seven databases, varying by combined score Q and mismatch k.

Table S5. Q=2.0e-02

| ***Group*** | ***k*** | ***Pax4*** | ***E-Prot-3*** | ***E-Prot-1*** | ***E-Mtab-513*** | ***E-Mtab-5214*** | ***Wang*** | ***E-Mtab-3358*** |
| --- | --- | --- | --- | --- | --- | --- | --- | --- |
| Infectious Disease | 0 | 0.0044+0.0044 | 0.0000+0.0000 | 0.0000+0.0000 | 0.0000+0.0000 | 0.0000+0.0000 | 0.0000+0.0000 | 0.0000+0.0000 |
| Healthy | 0 | 0.0256+0.0108 | 0.0000+0.0000 | 0.0000+0.0000 | 0.0000+0.0000 | 0.0000+0.0000 | 0.0000+0.0000 | 0.0000+0.0000 |
| Autoimmune Disease | 0 | 0.0461+0.0261 | 0.0000+0.0000 | 0.0000+0.0000 | 0.0000+0.0000 | 0.0000+0.0000 | 0.0000+0.0000 | 0.0000+0.0000 |
| Cancer | 0 | 0.0916+0.0187 | 0.0000+0.0000 | 0.0000+0.0000 | 0.0000+0.0000 | 0.0000+0.0000 | 0.0000+0.0000 | 0.0000+0.0000 |
| Infectious Disease | 1 | 0.0044+0.0044 | 0.0002+0.0002 | 0.0051+0.0051 | 0.0030+0.0030 | 0.0000+0.0000 | 0.0000+0.0000 | 0.0000+0.0000 |
| Healthy | 1 | 0.0262+0.0110 | 0.0029+0.0016 | 0.0215+0.0115 | 0.0239+0.0081 | 0.0000+0.0000 | 0.0000+0.0000 | 0.0082+0.0038 |
| Autoimmune Disease | 1 | 0.0471+0.0266 | 0.0090+0.0036 | 0.0517+0.0302 | 0.0380+0.0195 | 0.0000+0.0000 | 0.0000+0.0000 | 0.0169+0.0094 |
| Cancer | 1 | 0.0921+0.0188 | 0.0173+0.0040 | 0.0932+0.0199 | 0.0918+0.0171 | 0.0000+0.0000 | 0.0000+0.0000 | 0.0644+0.0136 |
| Infectious Disease | 2 | 0.0044+0.0044 | 0.0002+0.0002 | 0.0051+0.0051 | 0.0030+0.0030 | 0.0002+0.0002 | 0.0018+0.0018 | 0.0000+0.0000 |
| Healthy | 2 | 0.0262+0.0110 | 0.0030+0.0016 | 0.0223+0.0116 | 0.0246+0.0082 | 0.0007+0.0002 | 0.0093+0.0043 | 0.0085+0.0038 |
| Autoimmune Disease | 2 | 0.0472+0.0266 | 0.0090+0.0036 | 0.0522+0.0306 | 0.0387+0.0199 | 0.0013+0.0006 | 0.0430+0.0187 | 0.0170+0.0094 |
| Cancer | 2 | 0.0922+0.0188 | 0.0174+0.0040 | 0.0936+0.0200 | 0.0923+0.0171 | 0.0019+0.0004 | 0.0841+0.0171 | 0.0650+0.0136 |
| Infectious Disease | 3 | 0.0045+0.0044 | 0.0002+0.0002 | 0.0051+0.0051 | 0.0032+0.0030 | 0.0002+0.0002 | 0.0018+0.0018 | 0.0001+0.0000 |
| Healthy | 3 | 0.0263+0.0110 | 0.0030+0.0016 | 0.0223+0.0116 | 0.0247+0.0082 | 0.0007+0.0002 | 0.0098+0.0043 | 0.0086+0.0038 |
| Autoimmune Disease | 3 | 0.0473+0.0267 | 0.0090+0.0036 | 0.0522+0.0306 | 0.0389+0.0199 | 0.0012+0.0006 | 0.0437+0.0187 | 0.0172+0.0094 |
| Cancer | 3 | 0.0923+0.0188 | 0.0174+0.0040 | 0.0937+0.0200 | 0.0925+0.0171 | 0.0019+0.0004 | 0.0847+0.0171 | 0.0651+0.0136 |

Table S6. Q=1.0e-04

| ***Group*** | ***k*** | ***Pax4*** | ***E-Prot-3*** | ***E-Prot-1*** | ***E-Mtab-513*** | ***E-Mtab-5214*** | ***Wang*** | ***E-Mtab-3358*** |
| --- | --- | --- | --- | --- | --- | --- | --- | --- |
| Infectious Disease | 0 | 0.0199+0.0089 | 0.0000+0.0000 | 0.0000+0.0000 | 0.0000+0.0000 | 0.0000+0.0000 | 0.0000+0.0000 | 0.0000+0.0000 |
| Healthy | 0 | 0.0747+0.0189 | 0.0000+0.0000 | 0.0000+0.0000 | 0.0000+0.0000 | 0.0000+0.0000 | 0.0000+0.0000 | 0.0000+0.0000 |
| Autoimmune Disease | 0 | 0.1469+0.0439 | 0.0000+0.0000 | 0.0000+0.0000 | 0.0000+0.0000 | 0.0000+0.0000 | 0.0000+0.0000 | 0.0000+0.0000 |
| Cancer | 0 | 0.3400+0.0328 | 0.0000+0.0000 | 0.0000+0.0000 | 0.0000+0.0000 | 0.0000+0.0000 | 0.0000+0.0000 | 0.0000+0.0000 |
| Infectious Disease | 1 | 0.0212+0.0091 | 0.0024+0.0016 | 0.0232+0.0106 | 0.0159+0.0076 | 0.0000+0.0000 | 0.0000+0.0000 | 0.0099+0.0056 |
| Healthy | 1 | 0.0777+0.0194 | 0.0107+0.0029 | 0.0530+0.0186 | 0.0894+0.0185 | 0.0000+0.0000 | 0.0000+0.0000 | 0.0645+0.0155 |
| Autoimmune Disease | 1 | 0.1498+0.0445 | 0.0253+0.0061 | 0.1463+0.0506 | 0.0955+0.0324 | 0.0000+0.0000 | 0.0000+0.0000 | 0.1060+0.0307 |
| Cancer | 1 | 0.3440+0.0331 | 0.0667+0.0077 | 0.3768+0.0361 | 0.4005+0.0307 | 0.0000+0.0000 | 0.0000+0.0000 | 0.2753+0.0259 |
| Infectious Disease | 2 | 0.0215+0.0091 | 0.0027+0.0016 | 0.0250+0.0109 | 0.0173+0.0078 | 0.0003+0.0001 | 0.0120+0.0063 | 0.0107+0.0057 |
| Healthy | 2 | 0.0781+0.0194 | 0.0112+0.0029 | 0.0566+0.0190 | 0.0937+0.0189 | 0.0023+0.0006 | 0.0689+0.0171 | 0.0662+0.0156 |
| Autoimmune Disease | 2 | 0.1503+0.0445 | 0.0264+0.0062 | 0.1490+0.0514 | 0.0990+0.0330 | 0.0029+0.0006 | 0.1741+0.0414 | 0.1088+0.0309 |
| Cancer | 2 | 0.3444+0.0331 | 0.0672+0.0078 | 0.3806+0.0362 | 0.4042+0.0309 | 0.0101+0.0008 | 0.3695+0.0324 | 0.2787+0.0261 |
| Infectious Disease | 3 | 0.0216+0.0091 | 0.0028+0.0016 | 0.0253+0.0109 | 0.0178+0.0078 | 0.0003+0.0001 | 0.0132+0.0065 | 0.0112+0.0057 |
| Healthy | 3 | 0.0782+0.0194 | 0.0113+0.0029 | 0.0571+0.0190 | 0.0943+0.0189 | 0.0024+0.0006 | 0.0728+0.0173 | 0.0668+0.0156 |
| Autoimmune Disease | 3 | 0.1505+0.0445 | 0.0267+0.0062 | 0.1496+0.0514 | 0.0998+0.0330 | 0.0029+0.0006 | 0.1786+0.0418 | 0.1096+0.0309 |
| Cancer | 3 | 0.3445+0.0331 | 0.0674+0.0078 | 0.3810+0.0362 | 0.4048+0.0309 | 0.0101+0.0008 | 0.3725+0.0325 | 0.2792+0.0261 |

Table S7. Q=1.0e-05

| ***Group*** | ***k*** | ***Pax4*** | ***E-Prot-3*** | ***E-Prot-1*** | ***E-Mtab-513*** | ***E-Mtab-5214*** | ***Wang*** | ***E-Mtab-3358*** |
| --- | --- | --- | --- | --- | --- | --- | --- | --- |
| Infectious Disease | 0 | 0.0263+0.0098 | 0.0000+0.0000 | 0.0000+0.0000 | 0.0000+0.0000 | 0.0000+0.0000 | 0.0000+0.0000 | 0.0000+0.0000 |
| Healthy | 0 | 0.0817+0.0191 | 0.0000+0.0000 | 0.0000+0.0000 | 0.0000+0.0000 | 0.0000+0.0000 | 0.0000+0.0000 | 0.0000+0.0000 |
| Autoimmune Disease | 0 | 0.1885+0.0470 | 0.0000+0.0000 | 0.0000+0.0000 | 0.0000+0.0000 | 0.0000+0.0000 | 0.0000+0.0000 | 0.0000+0.0000 |
| Cancer | 0 | 0.4580+0.0379 | 0.0000+0.0000 | 0.0000+0.0000 | 0.0000+0.0000 | 0.0000+0.0000 | 0.0000+0.0000 | 0.0000+0.0000 |
| Infectious Disease | 1 | 0.0278+0.0100 | 0.0031+0.0016 | 0.0296+0.0115 | 0.0212+0.0084 | 0.0000+0.0000 | 0.0000+0.0000 | 0.0181+0.0074 |
| Healthy | 1 | 0.0854+0.0196 | 0.0120+0.0030 | 0.0580+0.0188 | 0.1044+0.0190 | 0.0000+0.0000 | 0.0000+0.0000 | 0.0702+0.0156 |
| Autoimmune Disease | 1 | 0.1914+0.0477 | 0.0399+0.0094 | 0.2035+0.0560 | 0.1357+0.0373 | 0.0000+0.0000 | 0.0000+0.0000 | 0.1435+0.0355 |
| Cancer | 1 | 0.4629+0.0382 | 0.0881+0.0087 | 0.5051+0.0416 | 0.5198+0.0339 | 0.0000+0.0000 | 0.0000+0.0000 | 0.3688+0.0299 |
| Infectious Disease | 2 | 0.0282+0.0100 | 0.0036+0.0016 | 0.0317+0.0118 | 0.0230+0.0086 | 0.0004+0.0001 | 0.0149+0.0066 | 0.0194+0.0075 |
| Healthy | 2 | 0.0860+0.0197 | 0.0127+0.0030 | 0.0623+0.0192 | 0.1097+0.0193 | 0.0030+0.0007 | 0.0815+0.0176 | 0.0725+0.0158 |
| Autoimmune Disease | 2 | 0.1922+0.0477 | 0.0410+0.0094 | 0.2063+0.0568 | 0.1394+0.0380 | 0.0033+0.0006 | 0.2300+0.0476 | 0.1463+0.0357 |
| Cancer | 2 | 0.4635+0.0382 | 0.0888+0.0087 | 0.5096+0.0418 | 0.5241+0.0341 | 0.0125+0.0009 | 0.4748+0.0353 | 0.3727+0.0301 |
| Infectious Disease | 3 | 0.0284+0.0100 | 0.0037+0.0016 | 0.0321+0.0118 | 0.0236+0.0086 | 0.0004+0.0001 | 0.0166+0.0069 | 0.0201+0.0075 |
| Healthy | 3 | 0.0861+0.0197 | 0.0129+0.0030 | 0.0629+0.0192 | 0.1106+0.0193 | 0.0031+0.0007 | 0.0863+0.0178 | 0.0733+0.0158 |
| Autoimmune Disease | 3 | 0.1924+0.0477 | 0.0413+0.0094 | 0.2072+0.0568 | 0.1404+0.0380 | 0.0033+0.0006 | 0.2345+0.0482 | 0.1473+0.0357 |
| Cancer | 3 | 0.4636+0.0382 | 0.0890+0.0087 | 0.5102+0.0418 | 0.5249+0.0341 | 0.0125+0.0009 | 0.4782+0.0354 | 0.3734+0.0301 |

***
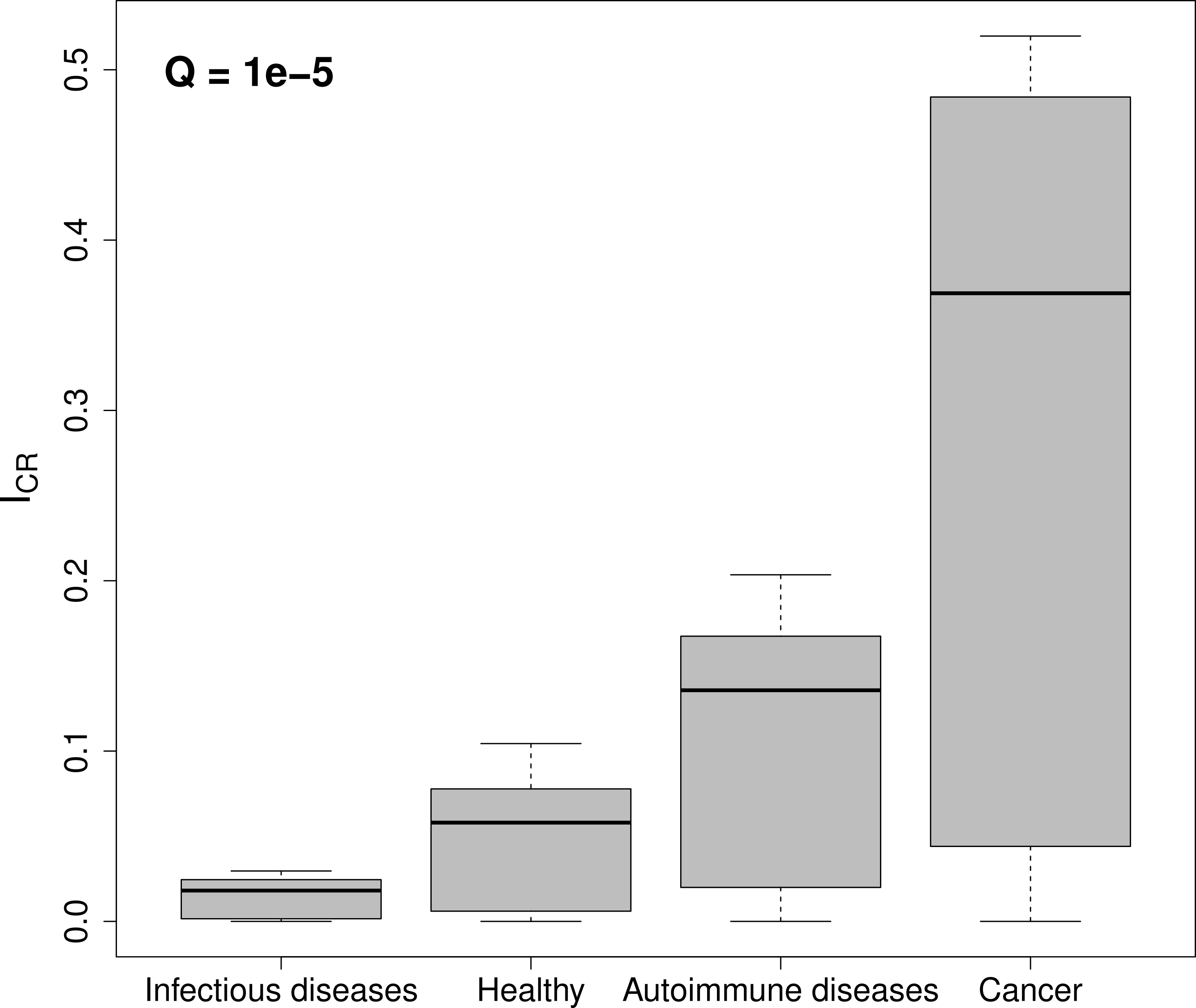
***

Fig. S2. The *<I_CR_*> indices for the four IEDB peptide groups (Table 2), obtained by averaging over the seven databases listed in Table 1. Thick black line: median; gray: the lower and the upper quartiles (25^th^ and 75^th^ percentiles); upper and lower whiskers: highest and lowest values. Q=1e-5, k=1.
